# Women's experiences of mistreatment during childbirth: A comparative view of home- and facility-based births in Pakistan

**DOI:** 10.1371/journal.pone.0194601

**Published:** 2018-03-16

**Authors:** Waqas Hameed, Bilal Iqbal Avan

**Affiliations:** 1 Research Scholar, Department of Statistics, University of Karachi, Sindh, Pakistan; 2 Department of Epidemiology and Population Health, London School of Hygiene and Tropical Medicine, London, United Kingdom; Tulane University School of Public Health and Tropical Medicine, UNITED STATES

## Abstract

**Introduction:**

Respectful and dignified healthcare is a fundamental right for every woman. However, many women seeking childbirth services, especially those in low-income countries such as Pakistan, are mistreated by their birth attendants. The aim of this epidemiological study was to estimate the prevalence of mistreatment and types of mistreatment among women giving birth in facility- and home-based settings in Pakistan in order to address the lack of empirical evidence on this topic. The study also examined the association between demographics (socio-demographic, reproductive history and empowerment status) and mistreatment, both in general and according to birth setting (whether home- or facility-based).

**Material and methods:**

In phase one, we identified 24 mistreatment indicators through an extensive literature review. We then pre-tested these indicators and classified them into seven behavioural types. During phase two, the survey was conducted (April-May 2013) in 14 districts across Pakistan. A total of 1,334 women who had given birth at home or in a healthcare facility over the past 12 months were interviewed. Linear regression analysis was employed for the full data set, and for facility- and home-based births separately, using Stata version 14.1.

**Results:**

There were no significant differences in manifestations of mistreatment between facility- and home-based childbirths. Approximately 97% of women reported experiencing at least one disrespectful and abusive behaviour. Experiences of mistreatment by type were as follows: non-consented care (81%); right to information (72%); non-confidential care (69%); verbal abuse (35%); abandonment of care (32%); discriminatory care (15%); and physical abuse (15%). In overall analysis, experience of mistreatment was lower among women who were unemployed (β = -1.17, 95% CI -1.81, -0.53); and higher among less empowered women (β = 0.11, 95% CI 0.06, 0.16); and those assisted by a traditional birth attendant as opposed to a general physician (β = 0.94, 95% CI 0.13, 1.75). Sub-group analyses for home-based births identified the same significant associations with mistreatment, with ethnicity included. In facility-based births, there was a significant relationship between women’s employment and empowerment status and mistreatment. Women with prior education on birth preparedness were less likely to experience mistreatment compared to those who had received no previous birth preparedness education.

**Conclusion:**

In order to promote care that is woman-centred and provided in a respectful and culturally appropriate manner, service providers should be cognisant of the current situation and ensure provision of quality antenatal care. At the community level, women should seek antenatal care for improved birth preparedness, while at the interpersonal level strategies should be devised to leverage women’s ability to participate in key household decisions.

## Introduction

In order to achieve Millennium Development Goal 5 (MDG), countries around the world focused considerable efforts on increasing the coverage of skilled birth delivery from 59% to 71%. As a result, maternal mortality fell by nearly 50% between 1990 and 2013[1] [1]. However, it is estimated that 303,000 women still die as a result of preventable causes during pregnancy and childbirth each year [2]. Most of these deaths occur in developing countries [1]. This unfinished agenda requires renewed efforts under the Sustainable Development Goal (SDG) agenda, with special focus on ensuring provision of quality maternal and newborn care that is both clinically appropriate and delivered in a respectful and dignified manner [3;4].

The World Health Organization (WHO) states that “every woman has the right to the highest attainable standard of health, which includes the right to dignified, respectful health care” [4]. Mistreatment during childbirth can potentially deter women from seeking medical care in future, leading to severely negative health implications [5;6]. Mistreatment during childbirth is not a new phenomenon. However, in recent years, it has gained widespread attention across the international community. This issue was formally prompted by human rights reports [7;8] and today, several studies [5;9–20] confirm that women across the globe frequently experience disrespectful and abusive care during childbirth.

This mistreatment can have immediate and long-term consequences: for example, denial of pain relief medication, episiotomy (without anaesthesia) and physical abuse can cause extreme pain and suffering [21]. It may also lead to adverse psychological effects such as re-traumatisation [22], post-traumatic stress symptoms, sleeping problems, poor self-rated health [12;21] and feelings of dehumanisation [23] that could result in a distorted body perception and fear of childbirth [23]. These encounters may deter women from seeking maternity care from health facilities [6]. As a result, they resort to home births, often relying on unskilled health professionals. This in turn increases the chances of maternal and neonatal morbidity and mortality [24].

Although several studies have attempted to define this phenomenon in the past [25–27], the issue requires more exploration due to the lack of consensus on the definition of mistreatment. The majority of research conducted on mistreatment during childbirth is from Africa [9–11;13–17;19] with minimal research from the South Asian region. To the best of our knowledge, there is no formal research available on this topic in Pakistan. Notably, the focus of recent research revolves heavily around childbirths conducted in facility-based settings. This focus is based on the argument that mistreatment is more prevalent in facility-based births due to the medicalisation [28–30] of childbirth and impersonal environment at facilities. This argument states that, in order for delivering women to regain their autonomy [28;31;32], they opt for home births, which are viewed as more women-centred and culturally appropriate [5;28]. However, there is no systematic assessment of home-based births related to the paradigm of respectful care.

Bearing in mind that the majority of low-income women in developing countries have home births [33], it is equally important to ascertain whether women giving birth at home receive respectful care.

In light of the dire need for evidence on disrespectful and abusive maternity care, this paper estimated the prevalence of mistreatment and types of mistreatment towards women giving birth in facility- and home-based settings. Secondly, the study examined the association between demographics (socio-demographic, reproductive history and empowerment status) and mistreatment, both in general and specific to the place of birth (home- or facility-based).

### Country context

Pakistan has an estimated population of 184 million [34] with 65% of the population residing in rural areas [35]. Pakistan has seen limited success on a select number of health issues; however, it has failed to achieve MDG 4 and 5 indicators [36;37]. Approximately one in four women do not receive any antenatal care (ANC), 52% give birth at home assisted by skilled birth attendants (SBA), and 62% receive a postnatal check-up (PNC) after delivery. Despite having a large public-sector healthcare system, only 15% of childbirths in Pakistan take place at public health facilities [35;38]. Factors impeding improved maternal and newborn health outcomes include structural and socio-cultural issues such as access, prohibitive cost and quality-of-care [36;38–41]. Quality of care issues range from providers’ technical incompetence to scarce resources and compromised interpersonal care at healthcare facilities [42–47].

## Methods

### Design and setting

We assessed women’s experiences of mistreatment as part of a larger population-based, cross-sectional survey conducted during April-May 2013 across 14 districts in Sindh Province (Jamshoro, Matiari, Ghotki, Shikarpur, Jacobabad, Khairpur, Qambar Shahdadkot, Larkana, Tando Alayar, Naushero Feroze, Karachi) and Punjab Province (Dera Ghazi Khan, Rahim Yar Khan, Rajanpur) in Pakistan. These districts are more marginalised than other districts in the selected provinces and were chosen because a new project on maternal and reproductive health was due to be implemented in these districts. Interviews regarding mistreatment during childbirth were conducted with women aged 15–49 who had had a live birth over the past 12 months. A total of 1,334 women met the eligibility criteria.

### Sampling

Data were collected in the catchment areas of 50 public and 50 private healthcare facilities that were selected for the larger maternal and reproductive health programme. These health facilities were located in remote areas and selected based on the criteria that they were situated at least 50 kilometres away from the district head quarter hospital and the service provider showed willingness to provide family planning services. Each health facility had a separate catchment population. Service coverage areas of 5–8 kilometres in radius were mapped around each health facility. The areas were classified into small clusters defined as ‘villages’ in rural settings and ‘blocks’ in peri-urban settings. Thereafter, one-quarter of the clusters were randomly selected within the catchment areas of each health facility. The population size for these clusters was estimated using crowd sourcing through at least five respondents residing in their respective clusters. These data were then matched with records available with government community health workers [48]. The probability proportional to size (PPS) technique was used to distribute the study sample across health facilities. The selection of households followed systematic sampling. The first household was selected using a ‘spin the bottle’ method at a major landmark within each cluster, followed by selecting every kth (approximately every 13^th^) household. Within each household, one woman meeting the criteria was invited to participate in the survey. If there was more than one eligible respondent in the household, enumerators selected the woman whose first name was closest to the alphabet ‘A’.

### Instrument development

The tool used to measure mistreatment during childbirth was developed using a two-phased approach. First, a comprehensive literature review was conducted on mistreatment during childbirth. From the literature, specific indicators were selected that were reviewed by experts with vast experience in the field of maternal health. The refined set of indicators was translated and pre-tested in two districts: Rawalpindi (Punjab Province) and Dadu (Sindh Province). Based on experts’ feedback and pre-test observations, some questions were rephrased and sequentially better arranged for easy administration. Importantly, questions pertaining to sexual abuse were excluded due to a high degree of sensitivity. In total, 24 indicators were finally retained based on seven behavioural types defined by Bowser and Hill [6]. These categories were reworked as follows: physical abuse, verbal abuse, right to information, non-consented care, non-confidential care, discrimination and abandonment of care. These indicators focused on the experiential aspect–i.e. women’s experiences of mistreatment–as defined by Freedman and colleagues [25]. The larger survey questionnaire was adapted from the 2006–07 Pakistan Demographic Health Survey (PDHS) instrument, which consisted of socio-demographic characteristics; information received on birth preparedness and essential postnatal/partum care; and women’s involvement in household decision making.

### Data collection and management

Questionnaires were translated into Urdu and Sindhi, and data were collected on paper forms by experienced female data collectors trained to administer the questionnaire. Data collectors conducted face-to-face interviews at participants’ homes to ensure privacy. Data were double-entered in Epi-data version 3.1.

### Measures

#### Outcome variables

The outcome variable for this study was experiences of mistreatment during childbirth based on 24 indicators classified into seven behavioural types. These classifications were divided as follows: (1) Physical abuse: (a) beating, (b) slapping, (c) push badly to change position, (d) pinch irritably; (II) Verbal abuse: (a) pass insulting or degrading comments, (b) harsh tone or shouting, (c) abusive language, (d) threatening for poor outcomes; (III) Non-consented care: (a) perform procedure without consent, (b) explain about the procedure to be used for delivery, (c) offer choices regarding births, (d) coercion to undergo caesarean section; (IV) Right to information: (a) share results/diagnosis of medical reports, (b) encourage to ask questions, (c) regularly share progress of childbirth; (V) Non-confidential care: (a) privacy during examination, (b) cover woman while taking to and from labour room, (c) assure woman for confidentiality of information, (d) women-provider conversation overheard by others (stranger, other patients, or non-medical staff); (VI) Abandonment of care: (a) abandon women during childbirth or afterward, (b) ignore while asking pain relief/medication, (c) delay birthing after deciding for operative procedure; and (VII) Discrimination: (a) denial of service due to ethnicity, (b) denial of service due to lack of money. Taking into consideration that participants may have previously given birth, they were instructed to answer these questions based on experiences during their most recent childbirth.

An ‘overall composite index’ was constructed for mistreatment along with indices for each type of mistreatment. Positively worded items were reverse coded for consistency where ‘1’ referred to ‘experienced mistreatment’, and ‘0’ to ‘not experienced’. Composite scores were calculated by adding the scores for each item. Total scores ranged from 0–24, where a higher score reflected a higher level of mistreatment. The same procedure was repeated for ‘individual composite indices’ for each type of behaviour.

#### Independent variables

Several variables were selected as factors that may be associated with mistreatment. These factors were categorised as follows: (I) Socio-demographics: women’s age, literacy, ethnicity, employment status and household poverty (using progress out of poverty tool) [49]; (II) Reproductive history: primigravida (whether experienced childbirths before), type of person who assisted with childbirth, place of birth (home- or facility-based), and education on birth preparedness and postnatal/partum care for the index pregnancy; and (III) Empowerment status: couples’ joint decision making (discrete composite index).

Under reproductive history, two separate variables were created for education: one for birth preparedness and one for postnatal/partum care. Women coded themselves as a ‘1’ if they were educated on any one of the seven essential birth preparedness elements (at least four antenatal visits, skilled birth attendant, transport arrangement, recognising danger signs, managing emergency, blood donor and keeping the baby warm) during pregnancy and ‘0’ if they had received none. A similar procedure was applied for postnatal/partum index, based on whether women were educated on: feeding of baby, mother’s nutrition, birth spacing (family planning), mother’s hygiene, baby’s hygiene and child immunisation.

The variable used to measure women’s empowerment was created using a method found in another study [50], and based on data collected on 15 indicators from an adapted framework proposed by Haque and colleagues [51]. Women were asked who is involved in making the following decisions: (1) where to go for medical care in the event of her illness; (2) where to take her children in the event of illness; (3) children’s education; (4) when to plan pregnancy; (5) number of children to have; (6) buying or selling of property; (7) small household expenditure (e.g. toothpaste, soap, crockery etc.); (8) major household expenditure (e.g. TV, refrigerator, furniture etc.); (9) expenditure on her clothes, cosmetics, jewellery etc.; (10) expenditure on medicines; (11) expenditure on children’s clothes; (12) where to spend earned money; (13) visiting relatives; (14) her employment outside the home; and (15) whether she leaves the house alone for medical help. A composite empowerment score for couples’ decision making was calculated and could range from 0–15, with a higher score reflecting that the respondent had a higher degree of decision-making power.

A poverty index was derived using the standard analytical approach as proposed by the Grameen Foundation [49].

### Ethics

The study protocol was reviewed and approved by the National Bioethics Committee (NBC), Pakistan (Ref: No. 4-87/NBC-109/RDC/1861).

The consent form included information about the purpose of interview, potential risks and benefits, a respondent’s right to choose to participate in the study, or to withdraw from it at any time, without reprisal, and to decline answering any question(s). Enumerators were asked to read the informed consent aloud to all participants and took written informed consent from each respondent prior to starting the questionnaire.

### Statistical analysis

A descriptive analysis of participants’ characteristics was completed using means and proportions. Linear regression analysis was applied with the intention of identifying individual level factors associated with reports of mistreatment rather than identifying underlying causes of mistreatment, which is impossible given the cross-sectional data. A block-modelling strategy [52] was applied to the full (home and facility birth) dataset whereby variables were entered into a model in groups, resulting in three different models: Model 1: examining the effect of socio-demographic variables; Model 2: including reproductive history for the index pregnancy; and Model 3: adding the status of women’s empowerment.

Moreover, one sub-group analysis was performed for each home- and facility-based birth with all variables in the model. Internal consistency for the 24 items on mistreatment–for each behaviour type, and for the 15 items on the women’s empowerment index–were tested using Kuder Richardson’s formula. Stata version 14.1 (StataCorp LP, Texas, United States) was used for all analyses, and p-values of 0.05 were considered statistically significant. Statistical weights were used to appropriately adjust the results based on representation of the population of all 14 districts. The population adjustment was made using the 2011 projected population for each district.

## Results

### Descriptive analysis

Table 1 presents the characteristics of women who were within one year postpartum. The majority of respondents were between 25 and 35 years old (55.1%) and had experienced several previous childbirths (84.0%). More than half (56.0%) were illiterate, about 70.0% were unemployed, and on average reported taking four household decisions jointly with their husbands regarding domestic affairs.

**Table 1:** Characteristics of the sample (n = 1,334).

Socio-demographics	%	Women’s empowerment*	%
Women’s age		Couple decision (out of 15)	
< 25 years	27.2	Mean (SD)	3.9 (4.8)
≥ 25 - < 35 years	55.1	**Reproductive history index pregnancy**	
≥ 35 years	17.8	Primigravida	
Ethnicity		Yes	16.0
Muhajir	12.1	No	84.0
Punjabi	36.4	Birth preparedness	
Baloch	14.5	Educated on at least one essential care (out of 7)	73.1
Pathan	4.9	None	26.9
Sindhi	32.1	Postnatal/partum care	
Women’s literacy		Educated on at least one essential care (out of 6)	47.3
Literate	44.0	None	52.7
Illiterate	56.0	Place of birth	
Women’s employment		Home	42.1
Yes	30.0	Health facility (public or private)	57.9
No	70.0	Type of birth attendant	
Household poverty index		General physician	40.5
Poorest	34.4	Mid-level provider	26.8
Middle	34.3	Traditional birth attendant	32.7
Wealthiest	31.3		

* Kuder-Richardson value for internal consistency = 0.94

In terms of ethnicity, approximately one-third of the women were Punjabi (36.4%) and Sindhi (32.1%); followed by Baloch (14.5%), Muhajir (12.1%) and Pathan (49%). Nearly three out of five women had given birth in a health facility, and the majority of births were assisted by doctors (40.6%), followed by traditional birth attendants (32.7%) and mid-level providers (26.8%). In addition, nearly three out of four women reported that they were educated on at least one element of birth-preparedness, while only 47.3% had received any education on postnatal/partum care.

**Fig 1:**
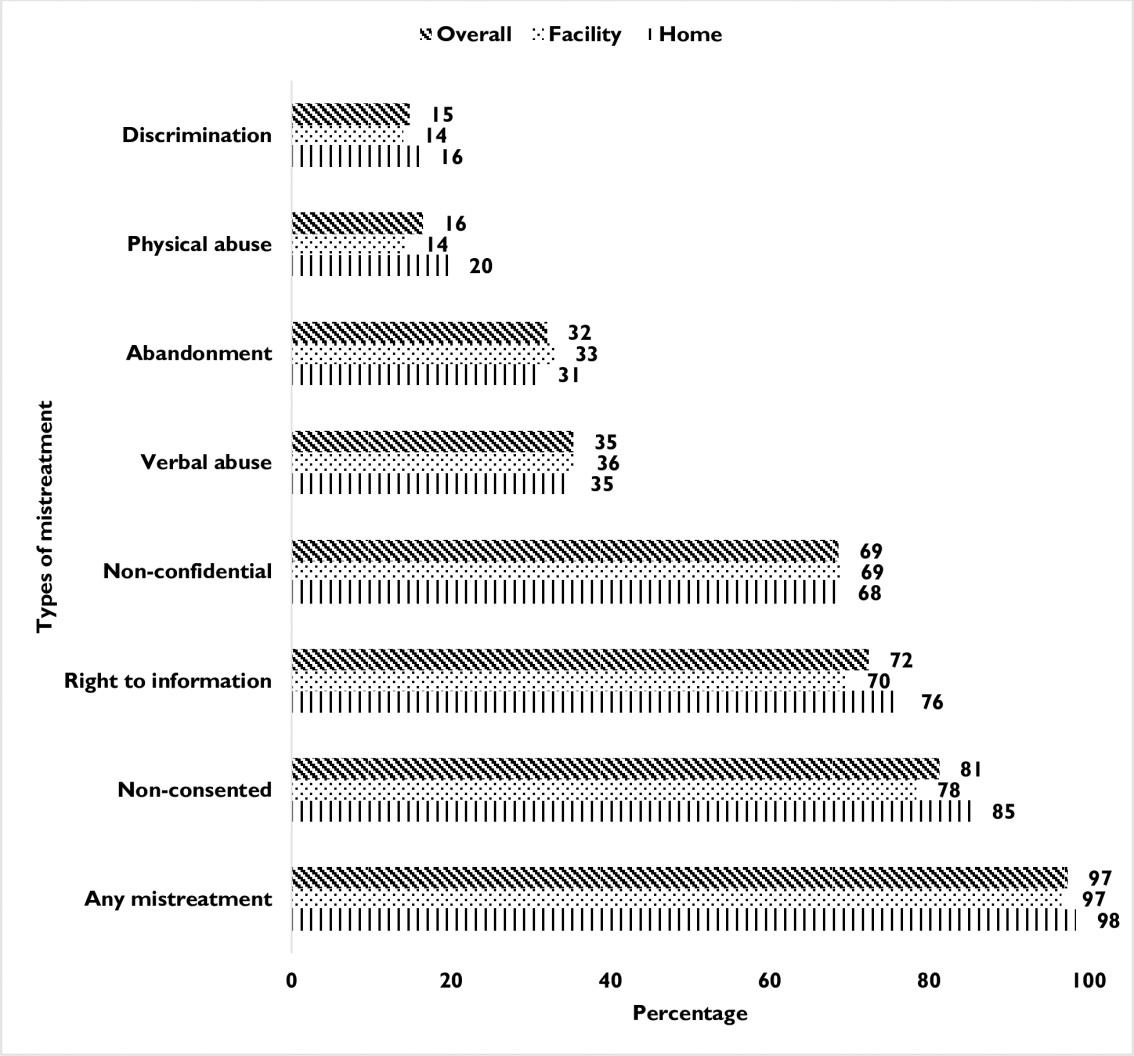
Prevalence of mistreatment by type and place of birth.

Fig 1. depicts experiences of self-reported mistreatment during facility- and home-based births. The prevalence of any mistreatment was universal (97%), and remained constant for both at-home and facility-based births. In terms of different manifestations of mistreatment, the most commonly reported was non-consented care (81%), followed by the right to information (72%) and non-confidential care (69%); while, nearly one-third reported experiences of verbal abuse (35%) and abandonment of care (32%). Overall approximately 15% of women reported physical abuse and discriminatory care. No substantial differences were observed in mistreatment during childbirth between home- and facility-based births.

**Fig 2:**
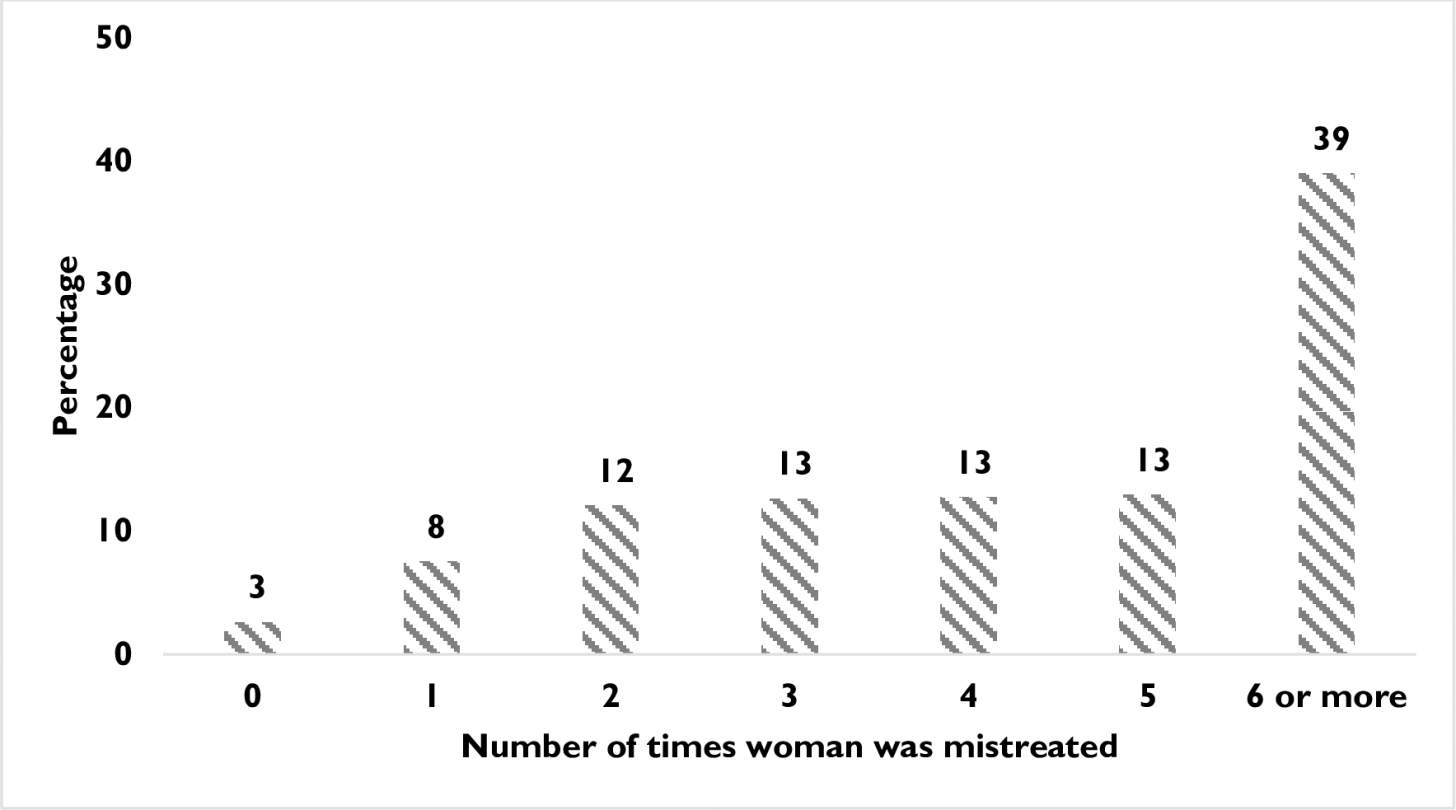
Prevalence of mistreatment care during childbirth.

Fig 2 illustrates the number of times self-reported mistreatment occurred at facility- and home-based births. Nearly two-fifths of the respondents reported being mistreated six or more times, while the majority of women reported being mistreated two to five times during childbirth.

Table 2 represents occurrences of mistreatment. On average, women experienced six different manifestations of mistreatment.

**Table 2:** Occurrence of mistreatment during childbirth by type.

Indicators	%	(95% CI)
**Any mistreatment**	97.4	(98.3,96)
**Average number of ways mistreated (SD)**	5.5	(5.2,5.8)
**Non-consented care (internal consistency = 0.17)**		
Any non-consented care	81.3	(84.3,77.8)
Perform procedure without consent	11.5	(9.3,14.2)
Didn’t explain about the procedure to be used for childbirth	46.7	(42.7,50.7)
Didn’t offer choices regarding childbirth	60.0	(55.9,63.9)
Coercion to undergo caesarean section	12.6	(10.2,15.6)
**Right to information (internal consistency = 0.68)**		
Any negligence in provision of information	72.4	(75.9,68.6)
Didn’t share results/diagnosis of medical reports	34.9	(31.3,38.8)
Didn’t encourage to ask questions	60.3	(56.3,64.2)
Didn’t regularly share progress of childbirth	47.2	(43.2,51.2)
**Non-confidential care (internal consistency = 0.31)**		
Any negligence in confidentiality	68.7	(72.3,64.8)
Didn’t maintain privacy during examination	14.1	(11.7,16.9)
Didn’t cover woman while taking to and from birthing area	37.4	(33.5,41.4)
Didn’t assure woman about confidentiality of information	23.4	(20.2,26.8)
Women-provider conversation overheard by others (stranger, other patients, or non-medical staff)	36.8	(32.9,40.8)
**Verbal abuse (internal consistency = 0.73)**		
Any kind of verbal abuse	32.5	(36.3,28.9)
Pass insulting or degrading comments	15.5	(12.7,18.6)
Harsh tone or shouting	19.1	(16.2,22.3)
Abusive language	16.4	(13.8,19.3)
Threaten for poor outcomes	10.8	(8.6,13.4)
**Abandonment of care (internal consistency = 0.55)**		
Any abandonment of care	32.1	(35.9,28.4)
Abandon women during childbirth or afterwards	20.2	(17.2,23.5)
Ignore while asking for pain relief/medication	16.1	(13.3,19.2)
Delay birthing after deciding for operative procedure	13.2	(10.8,16.1)
**Physical abuse (internal consistency = 0.76)**		
Any physical abuse	16.5	(19.6,13.7)
Beating	5.9	(4.4,8.0)
Slapping	6.7	(5.0,8.9)
Push badly to change position	8.3	(6.4,10.7)
Pinch irritably	10.7	(8.4,13.6)
**Discrimination (internal consistency = 0.72)**		
Any discrimination during care	14.8	(17.8,12.2)
Denial of service due to ethnicity	9.8	(7.7,12.4)
Denial of service due to lack of money	10.9	(8.7,13.4)

### Factors associated with mistreatment during childbirth

Linear regression results are presented in Table 3. Model 1 demonstrates associations between women’s socio-demographic characteristics and experiences of mistreatment during childbirth; Model 2 additionally takes into account indicators of women’s reproductive history for the index pregnancy; and Model 3 includes women’s empowerment status. The first three models were run on a full dataset. Ethnicity and women’s employment status showed significant associations with mistreatment (Table 3, Model 1). The relationship was still significant (in Model 2) after the inclusion of women’s reproductive history. However, with the inclusion of women’s empowerment index, the relationship between ethnicity and mistreatment became non-significant.

**Table 3:** Unadjusted and adjusted beta for mistreatment (discrete outcome) by sample characteristics.

Independent variable	Full dataset (facility + home delivery)						Sub-group analysis			
	Model 1		Model 2		Model 3		Adjusted coefficients for home-birth		Adjusted coefficients for facility-birth	
	Socio-demographics		Reproductive history		Women empowerment					
	β	95% CI	β	95% CI	β	95% CI	β	95% CI	β	95% CI
**Socio-demographics**										
Women’s age (Ref. = ≥ 35 years)										
≥ 25 - < 35 years	0.39	(-0.34, 1.12)	0.45	(-0.29, 1.19)	0.49	(-0.26, 1.23)	0.25	(-0.81, 1.32)	0.73	(-0.29, 1.75)
< 25 years	-0.02	(-0.84, 0.8)	0.09	(-0.77, 0.96)	0.15	(-0.72, 1.01)	-0.1	(-1.33, 1.13)	0.59	(-0.57, 1.75)
Ethnicity (Ref. = Muhajir)										
Punjabi	0.6	(-0.2, 1.41)	0.2	(-0.66, 1.06)	-0.05	(-0.9, 0.8)	0.28	(-1.23, 1.79)	0.14	(-0.95, 1.23)
Baloch	0.51	(-0.39, 1.41)	0.48	(-0.41, 1.36)	0.16	(-0.74, 1.07)	2.44*	(0.58, 4.3)	-0.65	(-1.73, 0.44)
Pathan	1.76*	(0.06, 3.46)	1.43	(-0.14, 3)	1.26	(-0.3, 2.81)	2.56*	(0.11, 5.0)	0.29	(-1.54, 2.12)
Sindhi	1.57***	(0.63, 2.51)	1.32*	(0.4, 2.25)	0.91	(0.0, 1.83)	1.83*	(0.04, 3.62)	0.49	(-0.57, 1.56)
Literacy status (Ref. = No)	0.37	(-0.26, 1)	0.3	(-0.33, 0.93)	0.24	(-0.38, 0.86)	0.12	(-0.87, 1.1)	0.38	(-0.4, 1.15)
Women’s employment (Ref. = Yes)	-1.16***	(-1.83, -0.49)	-1.1***	(-1.75, -0.45)	-1.17***	(-1.81, -0.53)	-1.03*	(-2.03, -0.02)	-1.08*	(-1.94, -0.22)
Household poverty (Ref. = Wealthiest)										
Middle	0.02	(-0.75, 0.79)	-0.09	(-0.86, 0.69)	-0.22	(-0.98, 0.55)	-0.48	(-1.84, 0.88)	-0.09	(-1.01, 0.82)
Poorest	-0.16	(-0.91, 0.58)	-0.4	(-1.17, 0.37)	-0.56	(-1.33, 0.2)	-0.87	(-2.1, 0.35)	-0.3	(-1.31, 0.72)
**Reproductive history for index pregnancy**										
Primigravida (Ref. = No)			0.04	(-0.78, 0.86)	-0.01	(-0.82, 0.8)	0.13	(-1.47, 1.72)	-0.39	(-1.34, 0.56)
Essential birth preparedness care (Ref. = Educated)			0.61	(-0.11, 1.33)	0.58	(-0.14, 1.3)	-0.21	(-1.15, 0.73)	1.3*	(0.16, 2.44)
Essential postnatal/partum care (Ref. = Educated)			-0.31	(-0.95, 0.32)	-0.32	(-0.95, 0.31)	0.27	(-0.84, 1.38)	-0.49	(-1.23, 0.25)
Type of birth attendant (Ref. = Doctor)										
Mid-level provider			0.8*	(0.08, 1.53)	0.68	(-0.04, 1.4)	1.05	(-0.66, 2.75)	0.73	(-0.1, 1.56)
Dai (Traditional birth attendant)			1.11*	(0.30, 1.92)	0.94*	(0.13, 1.75)	1.65*	(0.06, 3.24)	N/A	N/A
Women’s empowerment										
Couples’ decision—(Most empowered to least empowered) (range: 0–15)					0.11***	(0.06, 0.16)	0.11*	(0.03, 0.19)	0.11***	(0.05, 0.17)

Notes: N/A = Not applicable;

* p<0.05

**p<0.01

***p<0.001

We also found that women assisted by a mid-level provider (β = 0.80, 95% CI 0.08, 1.53) and traditional birth attendant (β = 1.11, 95% CI 0.30, 1.92) during childbirth reported higher levels of mistreatment compared to those assisted by a general physician.

The fully adjusted estimates in Model 3 revealed that, compared to working women, experiences of mistreatment was lower among women who were unemployed (β = -1.17, 95% CI -1.81, -0.53). Women’s empowerment was inversely associated with mistreatment (β = 0.11, 95% CI 0.06, 0.16)–in other words, less empowered women encountered higher levels of mistreatment. Additionally, with respect to who assisted with the childbirth, reports of mistreatment remained significantly higher among women who were assisted by traditional birth attendants as opposed to general physicians (β = 0.94, 95% CI 0.13, 1.75) whereas the difference between mid-level provider and general physician became insignificant (Table 3, Model 3).

The sub-group analysis for births conducted at home showed that experiences of mistreatment varied significantly by women’s ethnicity where women in Baloch (β = 2.44, 95% CI 0.58, 4.3), Pathan (β = 2.56, 95% CI 0.11, 5.0) and Sindhi (β = 1.83, 95% CI 0.04, 3.62) reported higher levels of mistreatment compared to Muhajir women. Similar to the full model (Model 3), the level of empowerment, employment status and type of person assisting in childbirth also showed statistically significant associations with mistreatment.

Results from births conducted in facility-based settings, showed that the same variables exhibited significant relationships with mistreatment. The only exception was that ethnicity became non-significant and higher reports of mistreatment were observed among women who had no prior education on birth preparedness compared with those who were more educated on this on birth preparedness.

## Discussion

This study aimed to estimate the prevalence of mistreatment women face during childbirth in Pakistan as well as the different types of mistreatment experienced. This study captured mistreatment during home births, unlike the majority of recent studies [5;12;13;16–20;53], which focused on institutional births. The overall prevalence of mistreatment was universal (97%), whereas prevalence for different types of mistreatment ranged from 15% to 81%. The experiences of mistreatment documented in this study were higher than studies from Africa [11;54;55]. Surprisingly, the current study found no differences in experiences of mistreatment between home- and facility-based births. These findings contradict the commonly accepted notion that home-based births are more humanised [5;28], as well as highlighting the prevailing issue of lack of supervision and accountability in home-based maternity care [56]. We suggest that attention should be paid to home-based births, which are often preferred by women who hope to receive culturally appropriate, supportive and less medicalised care [24].

As regards different types of mistreatment, violation of women’s right to information (72%), consented care (81%) and confidential care (69%) were the most frequently reported forms of mistreatment. Although these findings are similar to another study conducted in Ethiopia [11], it directly contradicts medical protocol that requires service providers to ensure confidentiality of care and to inform women about the necessity of–and potential risks from–the procedures in order to allow her to give her consent [57;58]. These manifestations may be more common because service providers lack an understanding of women’s rights [59], they may not consider these manifestations as major contributors to mistreatment [60], or these behaviours may have become normalised [25;61]. Specific to the issue of non-confidential care in facility-based settings, the presence of student interns and a large number of healthcare professionals may also reinforce this lack of privacy [62].

Literature identified that women are treated differently according to age, parity, ethnicity and socio-economic status [6;24]; however, none of these characteristics showed significant associations with mistreatment in our study. Measures that exhibited a strong relationship with mistreatment were proxies of women’s empowerment–the level of women’s involvement in household decision making and prior education on birth preparedness (see Table 3). While the impact of women’s empowerment on quality of life, health-seeking behaviours and maternal and child health outcomes is well known [63–65], our findings ascertain that provision of information and leveraging women’s ability to participate in key household decisions at the interpersonal level can also prevent them from being mistreated during childbirth. These findings are also consistent with a study in Tanzania, which found that birth-preparedness education (combined with other trainings) was effective in mitigating disrespectful maternity care [66].

The inverse relationship between women’s employment status with mistreatment is counter-intuitive and warrants further investigation. In addition, finding no statistically significant relationship between women’s literacy and mistreatment also seems counter-intuitive. The literature shows both positive [67] and negative [55] relationships between women’s education and mistreatment. To explain the significant relationship, some authors suggest that higher education increases expectations of better care and greater confidence to report mistreatment [55]. In addition, educated women are thought to be increasingly aware of their rights, and their increased level of self-confidence reduces the power differential between women and care providers, consequently reducing the likelihood of being mistreated [68]. We are of the view that the effect of education on mistreatment may have been diluted because our analyses accounted for women’s empowerment and level of awareness regarding birth preparedness, which are proxies for education.

In the full dataset (see Table 3, Model 3), we also found an association between mistreatment and the professional status of the care provider facilitating the delivery: traditional birth attendants treat women in a more disrespectful manner compared to general physicians. Notably, the magnitude further increased (from β = 0.94 to β = 1.65) in the sub-group analysis for births conducted at home. These findings may be attributed to a lack of professional training of the service providers and the fact that the care provided at home is more individualistic and not in compliance with any regulatory framework, as in the case of facility-based settings.

Results showed that women faced discrimination based on ethnicity in home-based births but not facility-based births. In Pakistan, almost all home births are conducted by traditional birth attendants [PDHS 12–13], and these care providers are not required to follow specific policies or codes of conduct as they are not regulated by the health system. As such, it seems plausible that these service providers may allow their own personal prejudices to play a factor in the care they provide their patients, as opposed to healthcare professionals in facilities who must follow certain guidelines.

It is necessary to acknowledge the limitations of this study: a) recall bias as the previous birth could have been at any point over the last year; b) occurrences of mistreatment may have been underreported, because some of the behaviours may be normalised [25]; c) some of the overestimation in reports of mistreatment way have been minimised by training the data collectors in non-judgemental interviewing techniques and asking probing questions to further ascertain mistreatment; d) the framework used in this study was based on the landscape analysis developed by Bowser and Hill [6], which has not yet been tested or validated, and focuses specifically on facility-based childbirth and more recently, experts have broadened the scope of mistreatment by incorporating systematic failures at the health system level that may directly or indirectly (via service providers) lead to mistreatment [24]; and e) this study uses the etic [69] approach to assess the experiential aspect of mistreatment while the subjective views of the respondents were not captured [25]. Research shows that certain behaviours that may be seen as disrespectful in some settings might be viewed as appropriate and acceptable under certain circumstances to ensure positive outcomes for the baby [61;70].

## Conclusion

To the best of our knowledge, this study is the first of its kind to present a comparative view of women’s experiences of mistreatment in facility- and home-based childbirth. The reports of mistreatment are overwhelmingly high during childbirth, irrespective of place of birth. Violation of a woman’s right to information, consented care and confidential care were the most prevalent forms of mistreatment, while physical and verbal abuse were reported less frequently.

In terms of risk factors, women’s employment status and their level of empowerment consistently demonstrated significant relationships with disrespectful care in all models. During home births, we found that women were also discriminated against based on their ethnicity and type of person assisting the childbirth. In facility-based births, on the other hand, women who had received no education on birth-preparedness reported higher experiences of mistreatment.

In order to promote care that is women-centred and provided in a respectful and culturally appropriate manner, service providers in all settings should be made aware of the current situation and measures should be taken to ensure provision of quality antenatal care. At the community level, women should seek antenatal care for improved birth preparedness while on the interpersonal level it is necessary to devise strategies to increase women’s ability to participate in household decision making with husband. Moreover, it is imperative to increase awareness and to develop a sense of entitlement to high-quality maternity care among both men and women. Future studies should integrate a mixed-methods approach to further enhance the understanding of mistreatment during childbirth in Pakistan.

### Disclaimer

The current study includes the collective views of authors, and does not necessarily represent the decisions or the stated policy of the London School of Hygiene and Tropical Medicine.
